# Exome sequencing analysis of murine medulloblastoma models identifies WDR11 as a potential tumor suppressor in Group 3 tumors

**DOI:** 10.18632/oncotarget.19642

**Published:** 2017-07-27

**Authors:** Lei Wei, Brian L. Murphy, Gang Wu, Matthew Parker, John Easton, Richard J. Gilbertson, Jinghui Zhang, Martine F. Roussel

**Affiliations:** ^1^ Department of Computational Biology, St. Jude Children’s Research Hospital, Memphis, TN, USA; ^2^ Department of Tumor Cell Biology, St. Jude Children’s Research Hospital, Memphis, TN, USA; ^3^ Pediatric Cancer Genome Project, St. Jude Children’s Research Hospital, Memphis, TN, USA; ^4^ Department of Developmental Neurobiology, St. Jude Children’s Research Hospital, Memphis, TN, USA; ^5^ Biostatistics and Bioinformatics, Roswell Park Cancer Institute, Buffalo, NY, USA; ^6^ Department of Molecular Oncology, Moffitt Cancer Center, Tampa, FL, USA; ^7^ Genomics England, Queen Mary University of London, London, UK

**Keywords:** medulloblastoma, whole-exome sequencing, WDR11, somatic mutations, mouse models

## Abstract

Mouse models of human cancers are widely used in cancer research, yet questions frequently arise regarding their faithfulness in recapitulating their human counterparts. To compare the somatic mutations of murine models with human medulloblastoma (MB), we performed whole-exome sequencing on 12 tumors representing three distinct medulloblastoma subgroups: Wnt, Sonic Hedgehog (Shh) and Group 3 (G3). In total, 64 somatic mutations were identified and validated, including 40 predicted to cause amino acid changes. After filtering and cross-species analysis with 366 human MBs from four independent studies, human orthologs for 16 of the 40 mouse genes were found to harbor non-silent mutations in human MB. Loss-of-function *Kmt2d* mutations detected in one mouse tumor was previously reported in 30 of 366 human MBs. In mice bearing G3 MB, one mouse succumbed to tumor burden at least 15 days earlier than other mice, raising the possibility that somatic mutations may have accelerated the tumorigenesis process. In this mouse tumor, four novel candidate genes harbored non-silent somatic mutations, *Lrfn2, Smyd1, Ubn2* and *Wdr11.* Extended survival was found in mice harboring mouse G3 overexpressing *WDR11* but not the other three genes. Genes in the KEGG WNT signaling pathway, including *Ccnd1/2/3*, *Myc* and *Tcf7l1*, were down-regulated in the transcriptome of G3 MB tumorspheres overexpressing WDR11, consistent with reduced tumor progression. In conclusion, we demonstrated that common spontaneous mutations were shared between human and murine models of MB suggesting similar molecular mechanisms of tumorigenesis, and identified WDR11 as a protein with tumor suppressive activity in G3 MB.

## INTRODUCTION

Genetically engineered mouse models (GEMM) provide a powerful *in vivo* system to test the role of genes mutated or overexpressed in human tumors [1]. Typically by overexpressing an oncogene or inactivating a tumor suppressor gene, normal murine cells can be transformed with histopathology similar to human cancers [2]. Despite the myriad of successful examples, mouse models have been frequently scrutinized with regards to their validity [3]. One key question is whether murine cancers and their human counterparts share similar tumorigenic processes which can be evaluated by comparing somatic mutational landscapes. Indeed, certain GEMMs have been found to harbor mutations in genes also mutated in human cancers [4, 5]. Next-generation sequencing (NGS) can comprehensively characterize the mutation landscape of cancers. However, NGS-based somatic mutation analysis in mice can be challenging due to complex mouse genetic background, especially when the matched normal DNA is not available [4].

Medulloblastoma (MB) is the most common malignant pediatric brain tumor that arises within the posterior fossa [6, 7]. Expression profiling of human MBs subdivides this cancer into four major molecularly distinct subgroups: Wingless (WNT), Sonic Hedgehog (SHH), Group 3 (G3) and Group 4 G4 [8-12]. Genetically engineered MB mouse models have been developed for Wnt [13], Shh [14-18] and G3 [19-22]. These subgroup-specific mouse models recapitulate the histopathology and gene expression profiles of their human counterparts [23] and can be utilized for screening and preclinical testing of therapeutics [24], which have been shown to increase the cure-rate of MB and quality of life of surviving patients [25-27]. However, very little is known about the role of additional somatic mutations involved in the process of tumorigenesis. To evaluate this in MB, we performed whole-exome sequencing (WES) to identify somatic mutations in 12 mouse tumors from 3 MB subgroup-specific murine models established at St. Jude Children’s Research Hospital: five Shh (MBS) [17], three Wnt (MBW) [13] and four G3 (MBM) [19]. A total of 64 somatic mutations were identified and experimentally validated, including 40 non-silent mutations predicted to cause amino acid changes. Survival data were available for the MB subgroup G3 mouse model - one mouse had a much shortened life than the other mice in the cohort. This mouse tumor had four genes with non-silent mutations, *Lrfn2, Smyd1, Ubn2* and *Wdr11.* We hypothesized that the mutations to these four genes may have facilitated tumor growth, and evaluated the function of each of these genes on G3 MB progression using *in vivo* systems.

## RESULTS

### Whole exome sequencing of tumor samples identified putative somatic mutations

**Figure 1:**
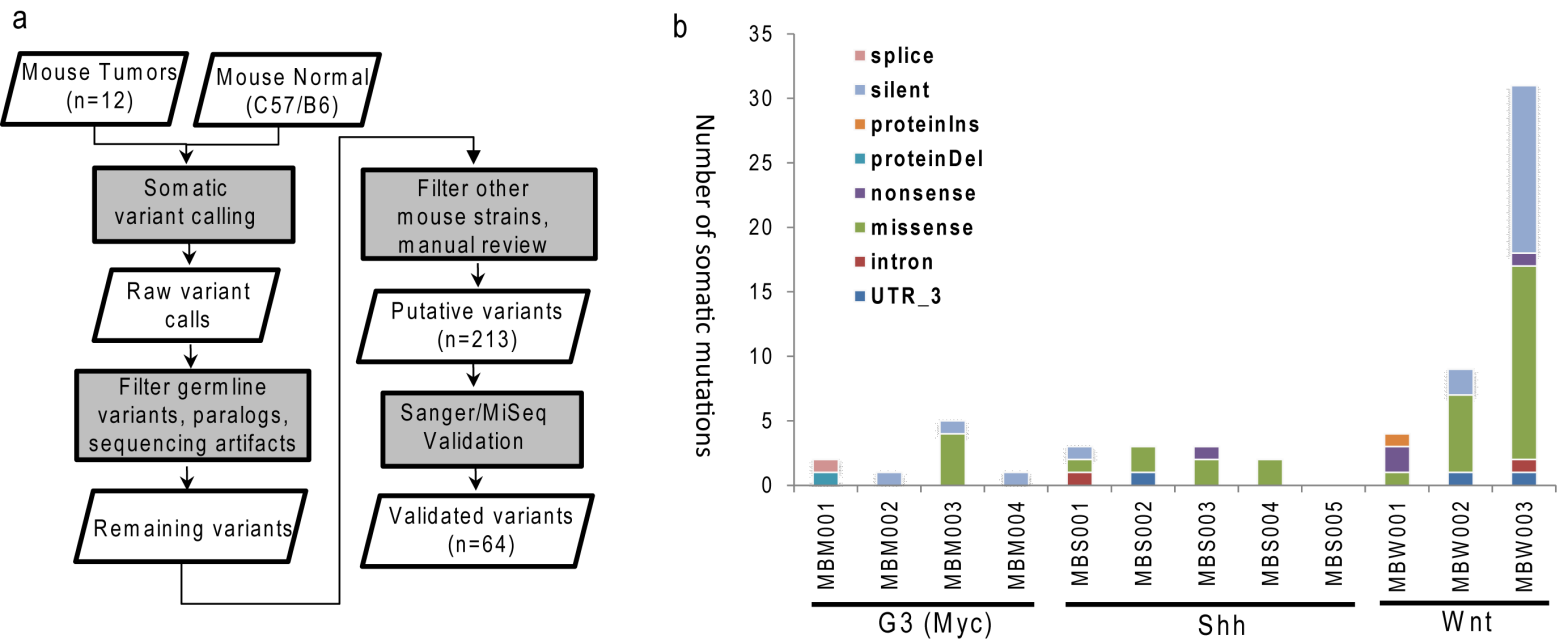
Identification of somatic mutations in MB mouse model **a.** Schematic workflow for somatic single nucleotide variations identification and validation; **b.** Numbers of mutations and events of validated somatic mutations in each MB mouse tumor. G3 (MBM), Shh (MBS), Wnt (MBW).

The murine MB models of three different subgroups were established as described previously [13, 14, 19, 28]. After whole-exome sequencing (WES), the reads were mapped to mouse genome (mm9) with at least 90% mapping rates across all samples (Supplementary Table 1). Most (11 of 12) samples had at least 80% coding bases covered by a minimum of 20x coverage. We did not retrospectively bank germline DNA needed for somatic mutation calling. To overcome this challenge, we went through extensive germline polymorphisms filtering process that removed both common mouse polymorphisms and rare variants found in other raw sequencing data of common laboratory mouse strains. The entire process of single nucleotide variations (SNVs) identification, filtering and validation is illustrated in Figure 1a. Specifically, the initial SNV calling was performed using Bambino [29] by comparing each tumor with publicly available normal sequences of C57BL/6 mouse strain [30]. With filtering and manual curation, 213 putative mutations remained. Amplicon sequencing by Sanger or MiSeq using tumor specimen and all available mice of common ancestors as control validated 62 somatic SNVs and two indels (Supplementary Table 2).

The overall mutation burden, 64 mutations in 12 mouse tumors or 5.3 mutations per tumor, is lower than previously reported for human MBs [8]. The number of somatic mutations per tumor ranged from 0 to 31, where the Wnt subgroup had higher burden than the Shh (*p*-value = 0.03) and G3 (*p*-value = 0.10) subgroups (Figure 1b). The final missense-to-silent mutation ratio was 1.7, and 40 mutations were predicted to cause changes in amino acid sequence. Due to the limited numbers of mice (3-5 per subgroup), no gene was recurrently mutated.

### Common non-synonymous mutations shared by MB mouse models and human MBs

To identify common mutations shared by mouse and human MBs, we compared the mutations identified from 12 mouse MBs to four independent human MB studies, consisting of a total of 366 MBs [8, 10, 11, 31]. Of the 40 mouse genes that carry amino-acid-changing mutations, 16 genes’ human orthologs were found to be mutated in human MBs. In seven genes, the exact type of mutation was found in the same subgroup of MB, including *BAI3*, *DOCK7*, *LRFN2*, *MLL2*, *MLL3*, *PIWIL4* and *WRD11* (Table 1). The most common mutations occurred in well-known MB genes *KMT2D* and *KMT2C* which contained 30 and 10 mutations in human MBs, respectively (Table 1). Remarkably, *Kmt2d* harbored a nonsense SNV predicted to cause loss of the SET domain which confers the methyltransferase activity [32, 33]. For comparison, the 366 human MBs harbored 30 *KMT2D* mutations, including 21 loss-of-function mutations (nonsense SNVs or frameshift indels) and nine missense mutations confirming the validity of the mouse models of MB. Specifically, 4 out of 48 *KMT2D* mutations occurred in WNT human MB subgroup, whereas one out of three WNT mice has a *Kmt2d* mutation (*p* = 0.015, hypergeometric test).

**Table 1:** Common non-synonymous mutations shared by mouse models and human MBs from four previous publications

gene	MB mouse (*n* = 12)	Lichter et al (*n* = 146)	Parsons et al (*n* = 53)	Pugh, Cho et al (*n* = 92)	Robinson et al (*n* = 75)
*BAI3*	WNT (1: M)	-	-	G3 (1: M)	WNT (1: M)
*DOCK7*	WNT (1: M)	WNT (1: M)	-	-	-
*GAPVD1*	WNT (1: M)	-	-	G4 (1: N)	-
*KIF14*	WNT (1: M)	G3 (1: M)	-	G3 (1: M)	-
*LRFN2*	G3 (1: M)	-	-	-	G3 (1: M)
*KMT2D*	WNT (1: N)	WNT (3: M2,N)	SHH,U,WNT (12: F6,M4,N2)	G3,G4,SHH (10: N5,F3,M2)	G3,SHH,U (5: F3,M,N)
*KMT2C*	WNT (1: M)	G3,G4 (2: M,N)	U,WNT (3: N3)	G3,G4 (4: M3,S)	U (1: N)
*MYO3A*	G3 (1: S)	-	-	SHH (1: M)	-
*OBSL1*	WNT (1: M)	-	-	SHH (1: M)	-
*OR2K2*	WNT (1: M)	-	-	-	SHH (1: N)
*PIWIL4*	G3 (1: D)	SHH (1: M)	-	-	G3 (1: M)
*SLCO5A1*	WNT (1: M)	-	-	G4 (1: M)	-
*SMYD1*	G3 (1: M)	-	-	-	WNT (1: M)
*TNXB*	WNT (1: M)	G3 (1: M)	-	G3 (1: M)	G3 (1: M)
*UBN2*	G3 (1: M)	-	-	-	SHH (1: M)
*WDR11*	G3 (1: M)	-	-	G3 (1: M)	-

First column - gene symbols; Columns 2 to 5 - mutations detected in the current MB mouse cohort, and in four previous publications. Detailed information includes: MB subgroup(s) where the mutations were detected; inside parenthesis ( ) represents the total number of mutations, and the breakdown by mutation class. M: missense, N: nonsense, S: splice variants, F: frameshift, D: in-frame deletion: a trailing number was added where more than one mutation were found in the medulloblastoma subgroup.

### Potential secondary driver mutation suggested by survival data

We addressed the overall survival of mice with G3 MBs (Supplementary Table 3). Among four mice in this group, MBM003, which had the highest mutation burden (Figure 1b), succumbed to tumor-burden at least 15 days earlier than the other mice. This led to the hypothesis that this mouse may harbor deleterious mutations which accelerated tumor progression. There were four non-silent mutations in this tumor, all missense SNVs: *Lrfn2* R656H, *Smyd1* R237Q, *Ubn2* Q549K and *Wdr11* I46T.

**Figure 2:**
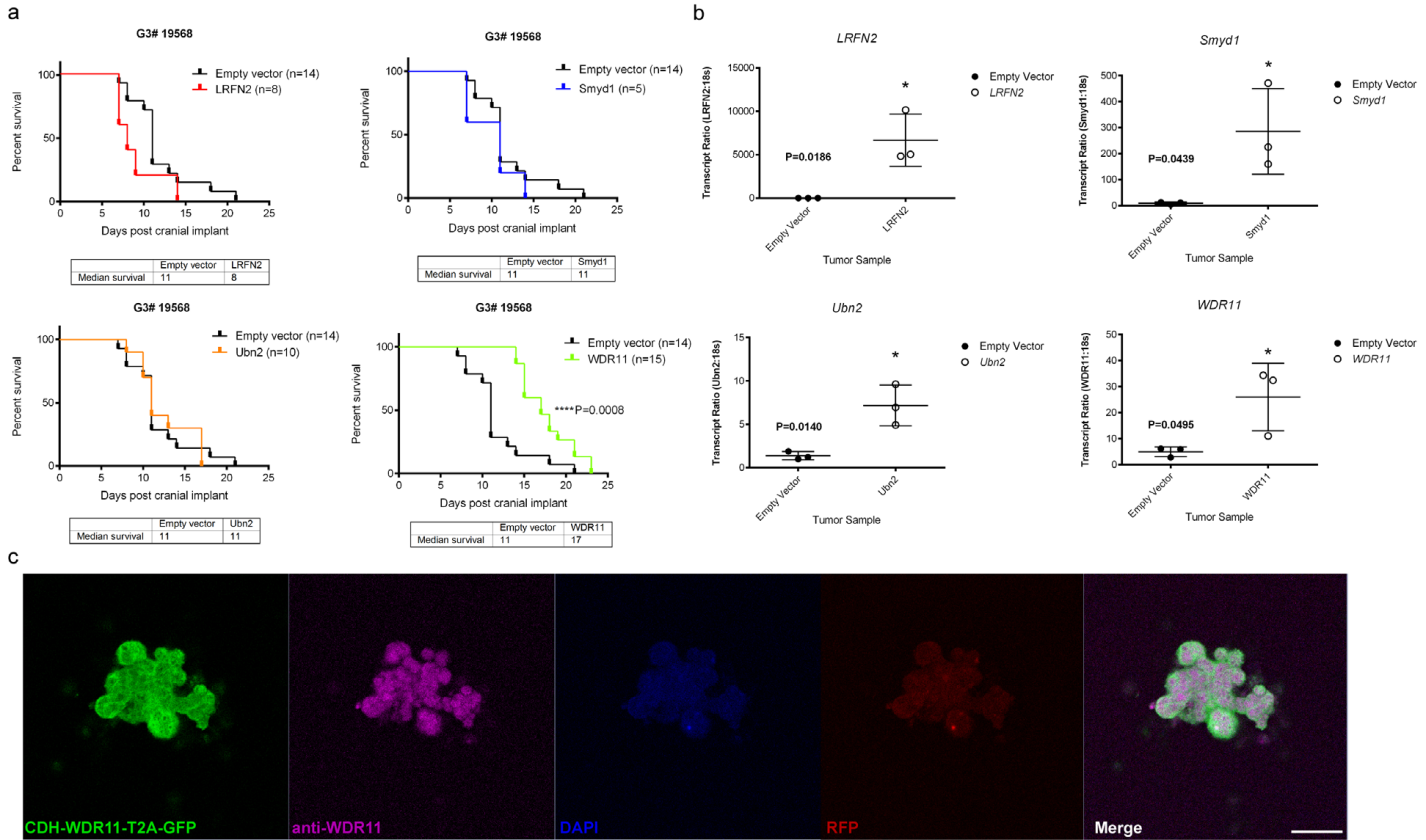
Functional study of genes mutated in mouse G3 MB **a.** Survival of mice bearing mouse G3 MB tumorsphere line # 19568 overexpressing each of the four wild type genes; *LRFN2* (red curve, top left panel), *Smyd1* (blue curve, top right panel), *Ubn2* (orange curve, bottom left panel), *WRD11* (green curve, bottom right panel), or an empty vector as control for each panel (black curves); **b.** Relative qRT-PCR transcript ratios of *LRFN2* (top left panel), *Smyd1* (top right panel), *Ubn2* (bottom left panel), and *WDR11* (bottom right panel) in G3 MBs overexpressing each wild type gene (o) or an empty vector (•); **c.** Protein expression detected by immunofluorescence in tumorspheres derived from secondary tumors following intracranial implants of tumorsphere line #19568 (myc-IRES-RFP) transduced with lentiviruses encoding *WDR11*. Tumorspheres were immunostained with anti-GFP (Green) to visualize CDH-WDR11-T2A-GFP, anti-WDR11 detected with AlexaFluor 647 (purple), anti-RFP (red) to visualize Myc expression and nuclei counterstained with DAPI (blue). Scale bar = 20 µm.

**Figure 3:**
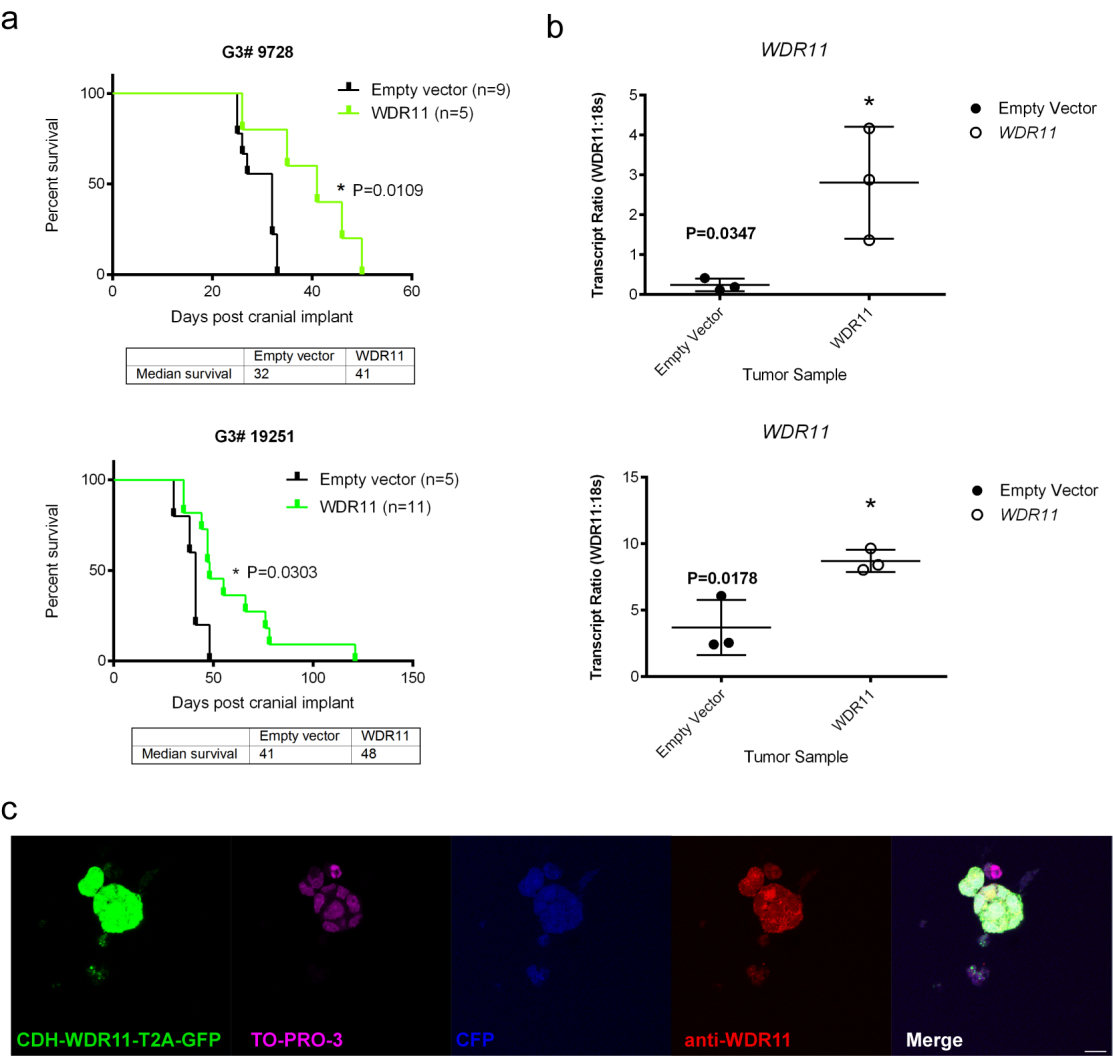
Survival of mice bearing mouse G3 MB overexpressing WDR11 in two additional G3 MB tumorsphere lines **a.** Survival of mice carrying mouse G3 MB tumorspheres # 9728 (top panel, green curve) or # 19251 (bottom panel, green curve) with enforced expression of *WDR11* compared to mouse G3 MB infected with an empty vector (black curves); **b.** Relative qRT-PCR transcript ratios of *WDR11* in secondary tumors overexpressing WDR11 (o) or infected with an empty vector (•) **c.** Protein expression detected by immunofluorescence in tumorspheres derived from secondary tumors following intracranial implants of tumorsphere line # 9728 (Myc-IRES-CFP) transduced with lentiviruses encoding *WRD11*. Tumorspheres were immunostained with anti-GFP (green) to visualize CDH-WDR11-T2A-GFP, anti-WDR11 detected by AlexaFluor 594 (red), anti-RFP and nuclei counterstained with TO-PRO^®^-3 (purple). Endogenous CFP (blue) was imaged to detect expression in Myc-IRES-CFP. Scale bar = 20 µm.

To test the potential tumor suppressor function of the four candidate genes, we used lentiviral transduction to enforce the expression of each of these wild type genes or an empty vector as control, in the mouse G3 tumorsphere line # 19568 prior to implantation of 100,000 unsorted tumor cells into the cortices of CD1 nu/nu mice (Figure 2). Enforced expression of *LRFN2* did not alter the survival of tumor-bearing mice (Figure 2a, upper left) despite its expression in resulting tumors (Figure 2b, upper left). Similarly, mice bearing mouse G3 MB tumor cells over-expressing *Smyd1* or *Ubn2* survived with similar latency compared to tumor cells transduced with empty vector (Figure 2a, upper right and lower left). In contrast, enforced expression of *WDR11* led to a significant increase in survival of tumor-bearing mice compared to mouse G3 tumorspheres infected with an empty vector (Figure 2a, lower right). Specifically, enforced expression of *WDR11* led to a 6-day increase in median survival of tumor-bearing mice. Analysis of these tumors showed increased *WDR11* transcript levels (Figure 2b, lower right). Protein expression was observed in tumorspheres from secondary tumors, measured by immunofluorescence (Figure 2c). These data suggested that in murine G3 MB, WDR11 had tumor suppressive activity since its enforced expression delayed tumor progression. To confirm these results, we over-expressed *WDR11* in two additional mouse G3 MB tumorsphere lines, # 9728 and # 19251. In these tumorsphere lines, over-expression of *WDR11* gene (Figure 3b) and protein (Figure 3c) also resulted in a significant increase in survival of *WDR11*-expressing tumor-bearing mice compared to controls (*p* < 0.05) (Figure 3a). These data identify WDR11 or its downstream effectors as potential targets for therapy in G3 MB.

### WDR11 overexpression leads to down-regulation of the WNT signaling pathway

**Figure 4:**
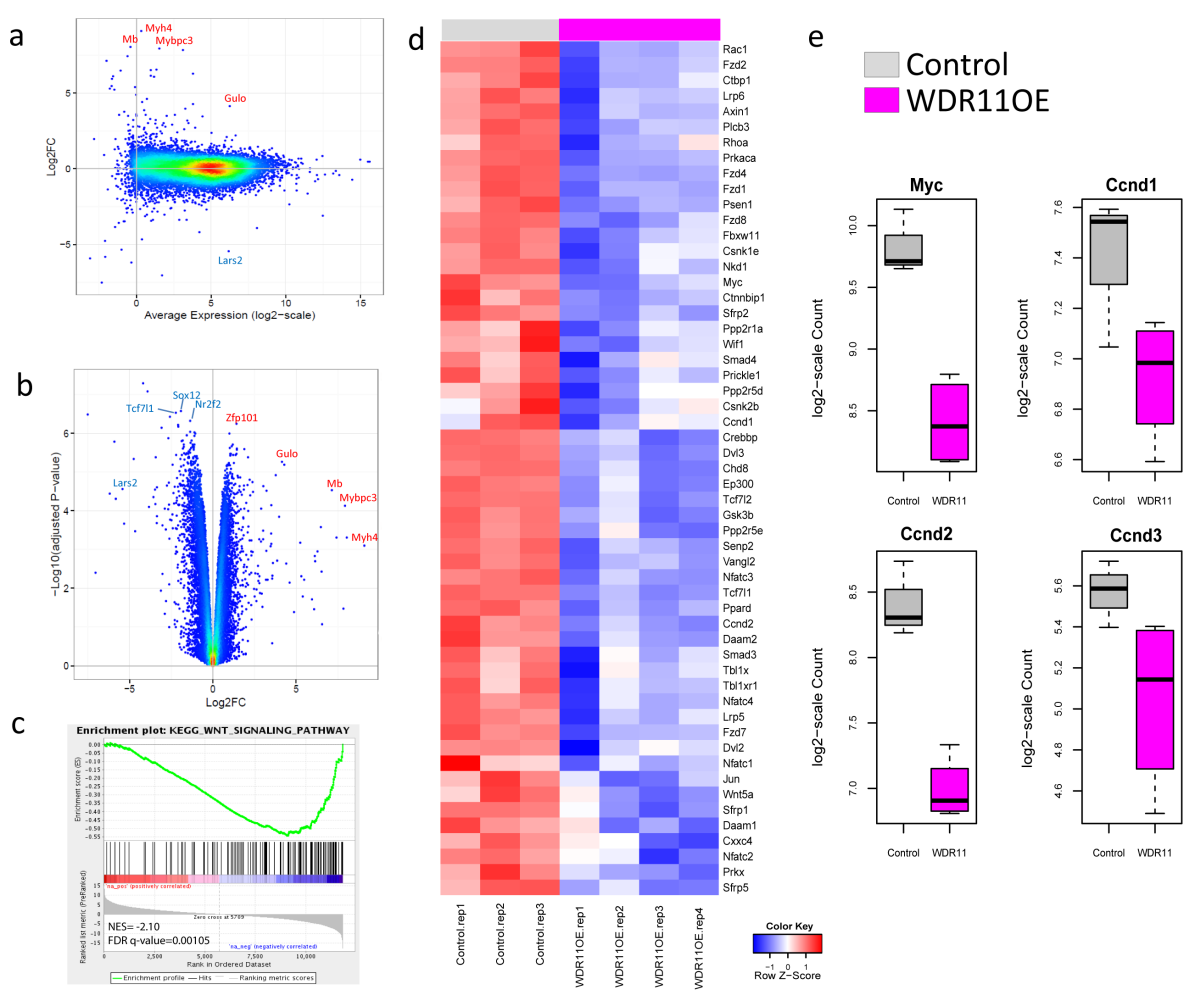
Over-expression of WDR11 induces down-regulation of WNT signaling **a.** and **b.** M-A (log2FC *vs* Average Expression) plot and volcano plot (-log10 adjusted p-value against log2FC) for the comparison of *WDR11* overexpressing mouse G3 MB cells *vs* those only carrying the empty vector; **c.**, WNT signaling pathway is down-regulated in the *WDR11* overexpressing cells through GSEA; **d.**, heatmap showing the expression of genes in the leading edge of the WNT signaling pathway; **e**., expression of *Myc*, *Ccnd1*, *Ccnd2* and *Ccnd3* in mouse Group3 MB with and without WDR11 over-expression.

To further understand the underlying mechanism by which *WDR11* overexpression contributed to the increased survival, we performed the whole transcriptome analysis on the mouse G3 tumors with and without *WDR11* overexpression (Figure 4). While tumors with enforced WDR11 expression induced a different overall gene expression pattern compared to G3 MBs, the biological replicates showed good concordance with some degree of variability (Supplementary Figure 1). Differential expression analysis identifies 624 genes down-regulated and 346 genes up-regulated at least 2-fold (Figure 4a, 4b, Supplementary Table 4, FDR < 0.05). Gene set enrichment analysis showed that genes in the KEGG WNT signaling pathways were down-regulated in tumor cells overexpressing WDR11 (Figure 4c, 4d, Supplementary Figure 2, Supplementary Table 5). The genes in the leading edge of the GSEA included *Myc*, *Ccnd1* and *Ccnd2* (Figure 4e), consistent with the delay in tumor development in mice harboring G3 MB overexpressing WDR11.

## DISCUSSION

In the past few years, human MBs have been extensively characterized at the molecular level by whole genome sequencing, exome sequencing, gene expression profiling and DNA methylation array [8, 10, 11, 31, 34]. These studies highlighted the paucity of somatic mutations found in MB and the complete absence of mutations in a large proportion of tumors, particularly in G3 and G4 MBs that represent the majority of tumors. The mutation burden from the current MB mouse models was even lower than that reported in human MBs. A possible explanation for the lower mutation burden is that mouse tumors are established in a *Trp53*-null background and either MYC overexpression in G3 MBs, *PTCH1* mutation in SHH MBs or *B-CATENIN* mutation in WNT MBs, all of which are found in corresponding human MB subgroups and sufficient to induce tumor development thus negating the requirement for additional driver mutations. Nevertheless, spontaneous mouse models of WNT and SHH and orthotopic models of G3 MB take 2-3 months to develop suggesting that additional mutations might be required to induce a full blown tumor.

One further question is whether the identified secondary mutations in mouse MBs match to the human MBs with similar background? In this study, we identified one *Kmt2d* nonsense mutation in the WNT subgroup. Remarkably, it has been reported that human WNT MBs also frequently harbor *KMT2D* nonsense or frameshift mutations, accounting for 16% of MB patients [30]. However, due to the limited sample size, we didn’t observe any shared mutations between human and murine SHH MBs. We previously analyzed and published chromosomal anomalies in orthotopic mouse SHH medulloblastoma derived from primary granule progenitors and found similar genomic alterations between mouse and human SHH MBs suggesting that these mouse models accurately model the human tumors [17, 35]. In future studies with additional samples and different types of data such as copy number, it will be interesting to evaluate other hallmark mutations.

Among the mutations identified in the mouse models of MB, epigenetic regulators appeared enriched compare to mutations in other biological pathways. In addition to *Kmt2d*, another mutated epigenetic histone modification gene was *Smyd1*, a histone methyltransferase that contains two known functional domains: MYND domain (myeloid translocation protein 8, Nervy, DEAF1) responsible for histone deacetylase-dependent transcriptional repression, and a SET domain [Su(var)3-9,enhancer-of-zeste,trithorax] that confers histone methyltransferase activity [32, 33]. *Smyd1* has been reported to regulate endothelial cells [36] and skeletal muscle [37] and to be mutated in an indolent B cell non-Hodgkin lymphoma [38] but not in brain tumors or in cerebellar development. This suggests that either *Smyd1* has a redundant function with other histone methyltransferases that compensate for its loss of function, or that this mutation is a passenger rather than a driver event. Indeed, in human MBs, inactivating mutations have been observed for *KTM2B, KTM2C, KTM2D* and *SETD2*, but not for other histone methyltransferases including *SMYD1* [8, 10, 11, 31, 34].

Interestingly, the murine G3 MB carrying a mutant Smyd1 had a shortened life-span (Supplementary Table 3). In addition to *Smyd1* mutation, this MB also carries mutation in three other genes (*Lrfn2*, *Ubn2* and *Wdr11*). These genes belong to families that were previously found to be involved in neuronal development or chromatin-remodeling [39-42]. Since these mutations were expected to be deleterious, this suggested that they may have tumor suppressor activity and that enforced expression of the wild type gene would suppress G3 MB progression. Indeed, enforced expression of wild-type *WDR11* (but not *LRFN2*, *Ubn2*, *Smyd1*) hindered G3 MB development, which is in-line with our hypothesis about its tumor suppressor activity. Over-expression of *WDR11* resulted in the down-regulation of WNT signaling pathway and regulators of G1 progression including *Myc* and D-type cyclins, Ccnd1,2 and 3 consistent with the delay in tumor development and increased survival.

*WDR11*, previously named *BRWD2* and then revised to *WDR11* due to the lack of bromodomains, is co-localized with *FGFR2* in tail-to-tail manner on chromosome 10q26 [43]. *WDR11* is a member of the WD-repeat gene family and ubiquitously expressed in normal brain and glial tumors [43]. The WD-repeat superfamily of proteins are found in all eukaryotes and implicated in a wide variety of functions including apoptosis, its loss of function would increase proliferation [44, 45]. While the exact function of WDR11 function is unknown, the proteins in that family have been found to be involved in many biological processes. Structural analysis revealed 12 WD domains in WDR11, nine of them form two β propellers. WDR11 co-localizes and interacts with EMX1, a homeodomain transcription factor that participates in the development of the central nervous system during early development of the brain [46], and missense mutations disrupting EMX1-binding in WDR have led to Kallman Syndrome, a genetic condition causing puberty failure [39]. The MB mouse mutation is a missense SNV that changes codon 46 from isoleucine to threonine. The site is evolutionarily conservative across mammals. *WDR11* was previously found to be mutated in one human MB [11] (Table 1). Another study also found WDR11 to be a tumor suppressor in glioblastoma (GBM) in which *WRD11* located on chromosome 10q26 [43], is inactivated in a balanced reciprocal translocation t(10;19) in a region that show frequent loss of heterozygocity (LOH) in GBMs. As the cost decreases, sequencing genetically engineered mouse tumors is becoming an effective approach to identify additionally acquired driver mutations, which might be shared by human and mouse tumors and help unveil the common molecular mechanisms of cancer [5]. However, somatic mutation calling remains a challenge, especially when the matched normal DNA is unavailable. Even for inbred CD1 *nu/nu* mouse strains, the real genetic background are often found to be surprisingly complex [4], which could not be resolved by simply filtering out common SNPs. In the current analysis, we developed a robust germline filtering pipeline using publicly available whole-genome sequencing data of 18 common laboratory strains. In addition to filtering out SNPs identified from these strains, we also checked the raw sequencing data for the weak evidence of any mutation calls being present, which helped remove germline SNPs in difficult regions such as the ones with low coverage or poor quality. Our extensive germline filtering process allowed us to reduce the mutation list to a manageable size with good validation rate. However, it is also possible that real mutations might have been accidentally excluded due to potential over-filtering. In the future, it is still preferred to have a matched normal sample available for somatic mutation calling.

In conclusion, our experiments highlight the differences in number and identify of the somatic mutations found between human and mouse tumors despite their similarity in pathology and gene expression pattern. We also identified Wdr11 as a putative tumor suppressor in Group3 MB.

## MATERIALS AND METHODS

### Animal husbandry

*[Ptch1+/-;Trp53-/-, Cdkn2c-/-]*, *[Ptch1+/-;Trp53-/-, Cdkn2c-/-]* (Uziel et al., 2005) and *[Blbp-Cre;Ctnnb1+/lox(Ex3); Tp53flx/flx]* (Gibson et al., 2010) mice develop spontaneous Shh and Wnt MBs. Mouse G3 MBs were generated by orthotopic transplantation of granule neuron progenitors (GNPs) purified from cerebella of 7 days old, P7, *Trp53-/-; Cdkn2c-/-* mice infected with Myc-encoding retroviruses into the cortices of 6-8- week-old CD-1 *nu/nu* mice (Charles River Laboratories), as previously described [19]. Mice were housed in an accredited facility of the Association for Assessment of Laboratory Animal Care in accordance with the NIH guidelines. The Institutional Animal Care and Use Committee of SJCRH approved all procedures in this study.

### Exome-capture and illumina sequencing

Genomic DNA was extracted from mouse Shh and Wnt spontaneously occurring tumors and G3 orthotopic MBs using the Qiagen DNAeasy kit. DNA samples were submitted to the Pediatric Cancer Genome Project Validation Lab for exome sequencing. Paired end sequencing reads have been mapped to mouse reference genome mm9 assembly. The mapping statistics was obtained from ‘samtools’ and the coverage statistics was obtained *via* an in-house coverage analysis pipeline (*covsum*) based on the RefSeq annotation of gene coding region.

### Identification of somatic mutations in mouse and mapping to human

Putative sequence variants including SNVs and indels were initially detected by running paired analyses using variation detection module of Bambino [29] with the following parameters: -min-flanking-quality 15 -min-alt-allele-count 2 -min-minor-frequency 0 -broad-min-quality 10 -mmf-max-hq-mismatches 15 -mmf-min-quality 15 -mmf-max-any-mismatches 20 -unique-filter-coverage 2 -min-mapq 1. A putative somatic sequence mutation was collected based on the following criteria: (1) Fisher’s exact test *P* value indicates that the number of reads harbouring the non-reference allele is significantly higher in tumour; (2) the non-reference allele frequency in tumour is > = 10%; Substitution variants are classified into four categories based on combination of their *P* value and sequence quality scores: High quality, high *P* value; high quality, low *P* value; low quality, high *P* value; low quality, low *P* value. *P* value refers to the *P* value of Fisher’s exact test comparing the distribution of the alternative allele in tumour and normal. High *P* value, *P* < 0.05; low *P* value, 0.05 < *P* < 0.10. A final review process re-maps and re-aligns the reads harbouring the non-reference allele to the reference genome to filter potential false positive calls introduced by mapping in repetitive regions and alignment artefacts. For putative somatic indels, the review process re-aligns all reads in tumour and normal at the indel site to a mutant allele template sequence constructed by substituting the wild-type allele with the indel. Presence of reads in normal sample that cover the mutant allele is considered a germline variant. Mouse gene symbols were converted to human ortholog gene symbols by using table “Orthology - Human *vs*. Mouse” from Mouse Genome Informatics (MGI), The Jackson Laboratory [47]. Gene symbols were mapped to geneID and RefSeq accession using “gene_info” and “gene2refseq” downloaded from NCBI (downloaded 04/29/2011). Afterwards, each mouse mutant variant was mapped to its human ortholog mRNA by running a local Blast to determine corresponding human mutation location.

To filter out germline SNPs, we first excluded any SNPs in public mouse germline SNP databases including: dbSNP build 128 [48], 18 common laboratory strains SNPs by The Wellcome Trust Sanger Institute [30], and Center for Genome Dynamics Mouse SNP Database from The Jackson Laboratory [49]. Afterwards, the remaining SNV calls were directly compared with the normal BAM files of the eighteen common laboratory strains by the Sanger Institute [30]. Putative mutations present in any of the eighteen strains were considered as non-somatic and excluded from further analyses. Remaining predictions were manually reviewed. Small insertions and deletions (indels) were identified in a similar manner. Overall, a total of 213 putative mutations, consisting of 207 SNVs and six Indels, were predicted and were sent for experimental validation.

### Validating the somatic mutations with non-tumor mice

Putative somatic SNVs and indels went through experimental validation using either amplicon-based MiSeq or Sanger sequencing (Figure 1a). Since the matched germline DNA was not available, we used all available mice of common ancestors for the validation experiment. Mutations present in any of these control mice were considered as non-somatic. After excluding one uncovered mutation in the validation experiment, the validation rate for the remaining 212 predictions was 30% (64/212). The highest cause of false predictions was germline events, which accounted for 41% (88/212). After all, we identified and validated 62 somatic SNVs and two indels (Supplementary Table 2).

### Subcloning of cDNAs and lentivirus production

The available murine and human cDNAs for the four genes of interest were purchased, amplified by polymerase chain reaction, and subcloned into pCDH cDNA cloning lentivectors (Systems Biosciences): human *LRFN2* (Accession No. BC142616, Clone ID 40147341; GE Dharmacon); mouse *Smyd1* (Accession No. BC076601, Clone ID 30609878; GE Dharmacon); mouse *Ubn2* (Accession No. BC051458, Clone ID 6468017; GE Dharmacon) and human *WDR11* (NM_018117; OriGene, SC113680). The homology between the human and mouse amino acid sequences of these genes were 95, 94, 90 and 94 percent, respectively. The cDNAs were amplified by PCR using the following primer sequences:. *LRFN2*_XbaI_F-TTCTAGAGTGACCAGACCATGGAGAC; *LRFN2*_NotI_R-TTGCGGCCGCGACCGTGCTCTCC ATCACCC; *Smyd1*_XbaI_F-ATTCTAGAGACTC TGAGATGACAATAGG; Smyd1_Not_R-TCGCGGCCGCGCTCCCCAGCCAC;

*Ubn2*_XbaI_F-TTTCTAGACAGAACAGTGGGGATGGCGGAGCCGCGC; *Ubn2*_NotI_R-TTGCGGCCGCCTGAGGTTTCCGTGGTAACTTAG; *WDR11*_XbaI_F-TACTCTAGAGCCACCGGGATGTTGCCCTACACA; *WDR11*_NotI_R-TTGCGGCCGCCTCTTCAATGGGTT

Wild type cDNAs of interest were subcloned into pCDH-EF1-MCS-T2A-copGFP (CD521A-1) and/or pCDH1-CMV-MCS-EF1-RFP (CD512B-1) using *XbaI* and *NotI*. The resulting lentiviral vectors were named: pCDH-EF1-LRFN2-T2A-copGFP, pCDH-EF1-Smyd1-T2A-copGFP, pCDH-EF1-Ubn2-T2A-copGFP, pCDH-EF1-WDR11-T2A-copGFP; pCDH1-CMV-LRFN2-EF1-RFP and pCDH1-CMV-Smyd1-EF1-RFP. High titer lentiviruses were produced transiently in 293T as previously described [50].

### Tumor sphere cultures, lentivirus infection, orthotopic transplants and tumor harvest

Mouse G3 MBs were grown as tumorspheres as previously described [19]. G3 MB tumorspheres # 19568 and #19251 were derived from tumors that developed in CD1nu/nu mice implanted with cerebellar granule neuronal progenitors transduced with MSCV retroviruses expressing Myc and the green fluorescent protein (GFP) from an internal ribosomal entry site (IRES) (Myc-IRES-GFP). G3 MB tumorsphere # 9728 was derived from tumors explanted from mice implanted with cerebellar granule neuronal progenitors transduced with MSCV retroviruses expressing Myc and the cyan fluorescent protein (CFP) from an internal ribosomal entry site (IRES) (Myc-IRES-CFP). Tumorspheres were derived from each of the three tumors and grown in N2, B27, EGF and FGF supplemented neurobasal medium (“complete tumorsphere medium”0, as previously described [19]. Tumorspheres were dissociated and infected six times over a 2-day period in virus collection media supplemented with N2, B27, EGF and FGF. The following day, infected tumorspheres were suspended in Matrigel (BD Bioscience, San Jose) at a concentration of 100,000 tumorspheres/5µl Matrigel and transplanted into the cortices of CD1-*nu/nu* mice, as described previously [19, 51]. Infection efficiency was analyzed using flow cytometry for RFP or GFP expression. In all cases, lentiviral infection efficiency ranged from 40-80%. After transplant of virus-infected tumorspheres, mice were examined daily for symptoms of sickness (doming of the head, ataxia or reduced activity). When mice became moribund, tumors were isolated and grown on a coverslip embedded in a 1:2 ratio (Matrigel: complete tumor sphere media) for 48 hours to allow for sphere formation and fixed in 4% paraformaldehyde for 15 minutes at room temperature followed by 3 rinses in PBS prior to immunofluorescence staining. Tumor chunks or cell pellets were also collected and stored at -80^◦^C.

### RNA extraction and quantitative real-time PCR

Total RNAs from tumorspheres and tumor chunks were extracted, reverse transcribed, and quantitative real-time PCR (qRT-PCR) for the following transcripts was conducted in accordance with manufacturer’s instructions using TaqMan RNA-to-Ct 1-step kit (ABI, 4392938) and TaqMan Gene Expression Assays [ABI, 43321182 with assay ID numbers of 4310893E (18s), Hs00608584_m1 (*LRFN2*), Mm00477663_m1 (*Smyd1*), Mm00723981_m1 (*Ubn2*), Hs00608584_m1 (*WDR11*)]. Relative levels of transcripts were quantified in triplicate. Ct values were normalized to *18s* and to parent tumor sphere culture (relative value = 1).

### Transcriptome sequencing and analysis

Total stranded RNA sequencing data were generated and mapped against mouse genome assembly NCBIM37.67 using the StrongArm pipeline described previously. Four murine G3 tumor samples overexpressing human WDR11 and three mouse G3 MB tumors expressing the empty vector control were characterized. The gene level quantification values were obtained with HT-seq [52] based on GENCODE annotation and normalized by TMM method with ‘EdgeR’ package [53]. Differential expression analysis was performed in ‘voom’ method in R ‘limma’ package [54]. Gene set enrichment analysis was carried out using GSEA with MSigDB [55].

### Immunofluorescence and confocal microscopy

Tumorspheres were stained with antibodies to LRFN2 (Millipore, 1:250), SMYD1 (abcam, 1:250), UBN2 (Abgent, 1:250) or WDR11 (abcam, 1:250) and GFP or RFP (Abcam, 1:500). Tumorspheres were nuclear counterstained using DAPI or TO-PRO^®^-3 and immunostained cells were imaged using confocal microscopy. Images were acquired using a Zeiss 510 Meta point scanning confocal/multiphoton microscope equipped with 20X dry [0.8 numerical aperture (NA)], 40X oil immersion (1.3 NA) and 60X oil immersion (1.4 NA) objectives. The Argon single photon excitation laser was used to detect GFP at 488 nm, while the Helium/Neon laser detected RFP and AF594 at 543 nm and AF647 and TO-PRO^®^-3 at 633 nm. A femtosecond pulsed laser was used to excite and detect DAPI and CFP. 1024 x 1024 pixels optical sections were acquired (averaged four times) using the Zen 2009 software (version 5.5.0.433).

### Statistical analysis

The Kaplan-Meier method was used to calculate the significance of mouse survival. Statistical analyses were performed in the GraphPad Prism software version 6.0.

## SUPPLEMENTARY MATERIALS FIGURES AND TABLES













## Abbreviations

DNA: Deoxyribonucleic Acid
WNT: Wingless
SHH: Sonic Hedgehog
GSEA: Gene Set Enrichment Analysis
MB: medulloblastoma
G3: Group 3
GEMM: genetically engineered mouse models
NGS: Next-generation sequencing
WES: Whole-exome sequencing
MBS: Sonic hedgehog subgroup-specific murine medulloblastoma model
MBW: Wnt subgroup-specific murine medulloblastoma model
MBM: G3 subgroup-specific murine medulloblastoma model
mm9: University of California Santa Cruz mouse genome browser mm9 assembly
SNVs: Single nucleotide variations
cDNA: Complementary DNA
RFP: Red fluorescent protein
CFP: Cyan fluorescent protein
GFP: Green fluorescent protein
N2: N-2 supplement
B27: B-27 serum-free supplement
EGF: Epidermal growth factor
FGF: Fibroblast growth factor
PBS: Phosphate buffered saline
RNA: Ribonucleic acid
qRT-PCR: Quantitative real-time polymerase chain reaction
DAPI: 4’,6-Diamidino-2-Phenylindole (CAS name: 1H-Indole-6-carboximidamide, 2- [4-(aminoiminomethyl)phenyl]-, dihydrochloride)
TO-PRO^®^: CAS name: Quinolinium,4- [3-(3-methyl-2(3H)-benzothiazolylidene)-1-propenyl]-1- [3-(trimethylammonio)propyl]-, diiodide
NA: Numerical aperture
