# Supplementary material for: Exome sequencing analysis of murine medulloblastoma models identifies WDR11 as a potential tumor suppressor in Group 3 tumors
Source: Oncotarget. 2017 Jul 27;8(39):64685–97. doi: 10.18632/oncotarget.19642 (PMC5630286; doi:10.18632/oncotarget.19642)
Supplement: Supplementary file 1 [file oncotarget-08-64685-s001.pdf]

Exome sequencing analysis of murine medulloblastoma models identifies WDR11 as a potential tumor suppressor in Group 3 tumors

SUPPLEMENTARY MATERIALS

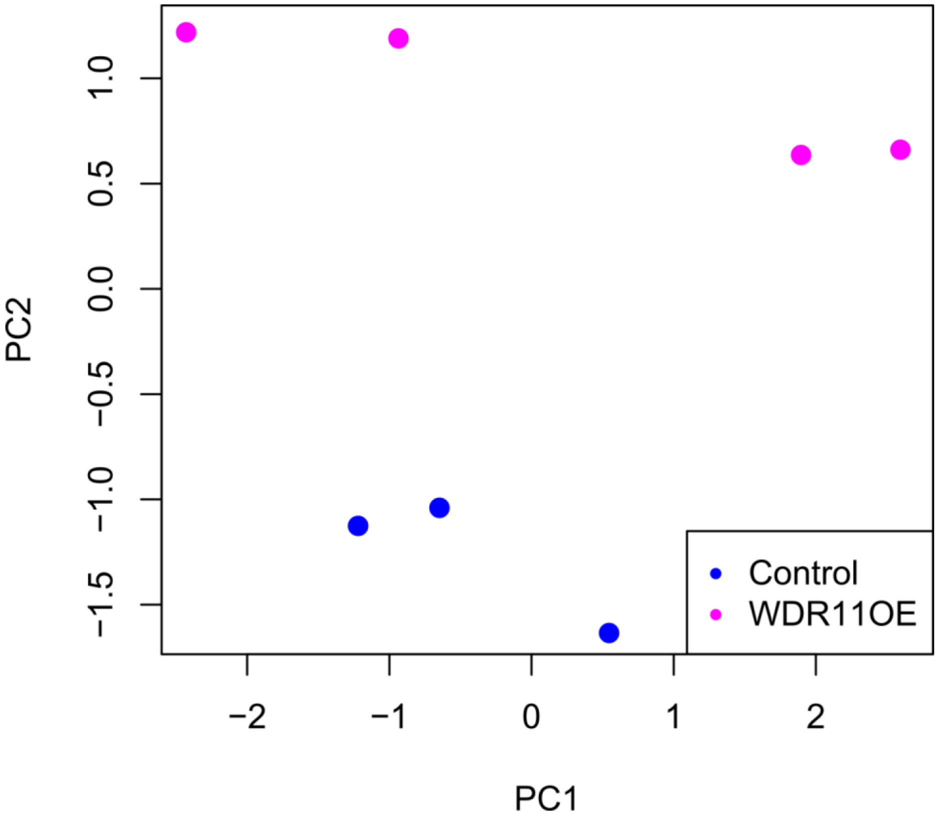

Supplementary Figure 1: Principal component analysis of the gene expression of mouse Group3 MBs with or without enforced expression of WRD11 by the RNA-Sequencing.

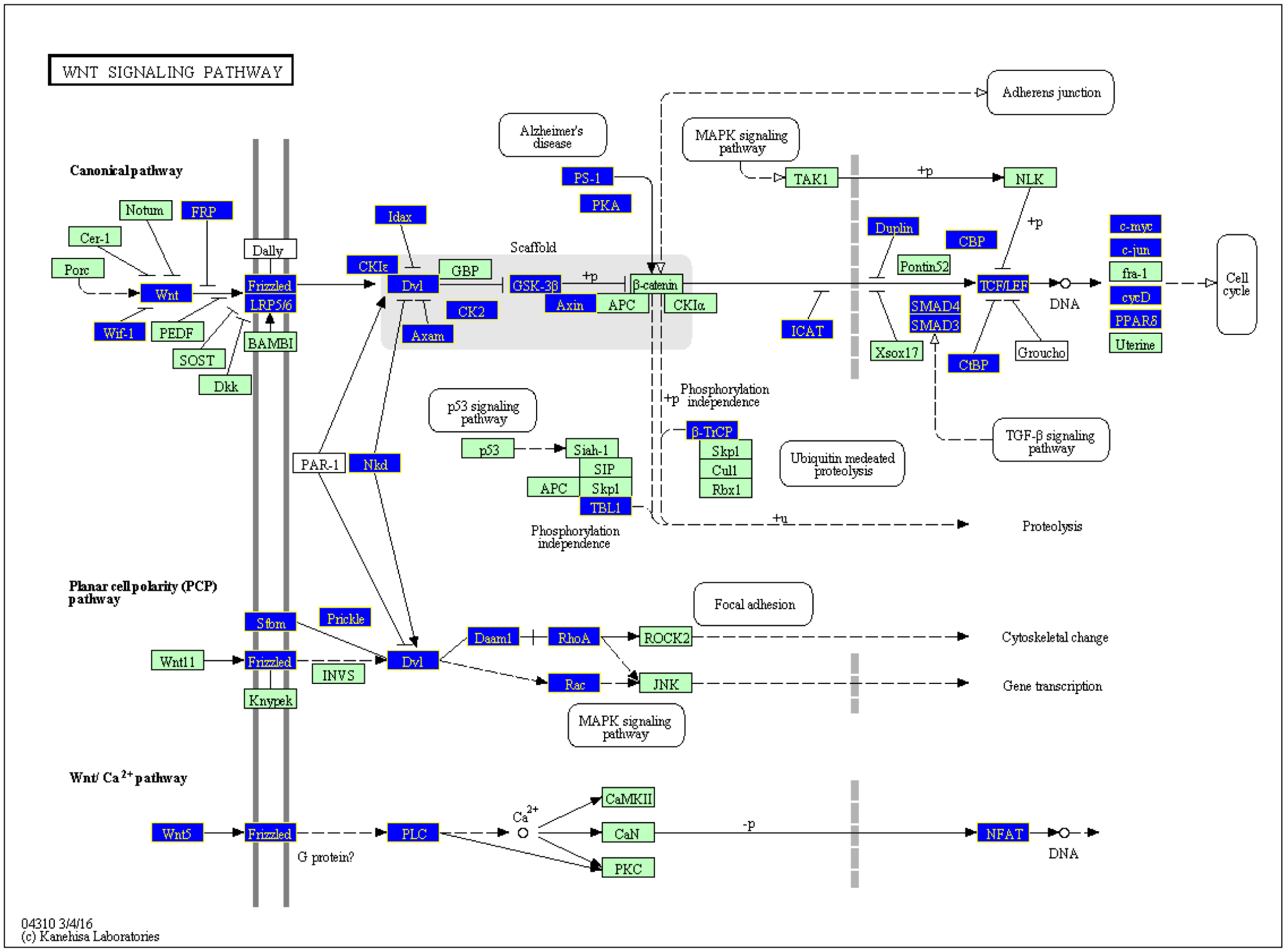

Supplementary Figure 2: Global down-regulation of the WNT signaling pathway. Downregulated genes were highlighted in blue boxes using KEGG Mapper.

For Supplementary Tables 1-5, see the appropriate links on the Supplementary Files page
